# Avipoxviruses: infection biology and their use as vaccine vectors

**DOI:** 10.1186/1743-422X-8-49

**Published:** 2011-02-03

**Authors:** Simon C Weli, Morten Tryland

**Affiliations:** 1 National Veterinary Institute, Ullevålsveien 68, N-0106 Oslo, Norway; 2 Section of Arctic Veterinary Medicine, Department of Food Safety and Infection Biology, Norwegian School of Veterinary Science, Stakkevollveien 23, N-9010 Tromsø, Norway; 3 GenØk-Centre for Bio-safety, The Science Park, Breivika, PO Box 6418, N-9294 Tromsø, Norway

## Abstract

Avipoxviruses (APVs) belong to the *Chordopoxvirinae *subfamily of the *Poxviridae *family. APVs are distributed worldwide and cause disease in domestic, pet and wild birds of many species. APVs are transmitted by aerosols and biting insects, particularly mosquitoes and arthropods and are usually named after the bird species from which they were originally isolated. The virus species Fowlpox virus (FWPV) causes disease in poultry and associated mortality is usually low, but in flocks under stress (other diseases, high production) mortality can reach up to 50%. APVs are also major players in viral vaccine vector development for diseases in human and veterinary medicine. Abortive infection in mammalian cells (no production of progeny viruses) and their ability to accommodate multiple gene inserts are some of the characteristics that make APVs promising vaccine vectors. Although abortive infection in mammalian cells conceivably represents a major vaccine bio-safety advantage, molecular mechanisms restricting APVs to certain hosts are not yet fully understood. This review summarizes the current knowledge relating to APVs, including classification, morphogenesis, host-virus interactions, diagnostics and disease, and also highlights the use of APVs as recombinant vaccine vectors.

## Introduction

Avipoxviruses (APVs) are among the largest and most complex viruses known. APVs belong to the *Chordopoxvirinae *subfamily of the *Poxviridae *family [1]. They infect and cause diseases in poultry, pet and wild birds of many species which result in economic losses to the poultry industry. Infections have also been reported in a number of endangered species or species in captive-breeding recovery programs [2-4]. APVs are transmitted via biting insects and aerosols and are usually named on the basis of the bird species from which the virus was first isolated and characterized [4]. The disease, which is characterized by proliferative lesions of the skin and diphtheric membranes of the respiratory tract, mouth and oesophagus has been described in avian species [4,5]. Although APV infections have been reported to affect over 232 species in 23 orders of birds [6], our knowledge of the molecular and biological characteristics of APV is largely restricted to fowlpox virus (FWPV) and canarypox virus (CNPV) for which full-genome sequences are available [7,8]. Currently, only ten avipoxvirus species are listed under the genus by the International Committee on Taxonomy of Viruses (ICTV) [1]; Table 1. Thus, it is safe to assume that many APVs have yet to be characterized. Recombinant APVs have been evaluated for use as vaccine vector candidates against infectious diseases [7,9]. APV-vectored vaccines are already in use in veterinary medicine [10-14], and it is likely that such vaccines will also be used against human diseases in the future. This fact emphasizes the need to learn more about the molecular characteristics of APVs, which underpins the development of safe APV-vectored recombinant vaccines. This review summarizes current knowledge of APVs as avian pathogens, including classification, morphogenesis, host-virus interactions, diagnosis, as well as issues relevant to their use as recombinant vaccine vectors.

**Table 1:** Members of the genus *Avipoxvirus *and their host spectrum

Type species	Hosts	Latin names	Mode of infection	Disease confirmation	References
Fowlpox virus^1a^	Chicken	*Gallus gallus*	E, N	HP, CAM, EM, PCR/Seq	[7,21,27,113]
Canarypox virus^1b^	Canary (several species)	*Serinus canarius*	E, N	HP, CAM, EM, PCR/Seq	[8,17,114,115]
Juncopox virus^1^	Slate coloured Junco	*Junco hyemalis*	E	GE	[48]
Mynahpox virus^1^	Greater hill mynahs	*Gracula religiosa*	E, N	HP	[116]
Psittacinepox virus^1^	Parrot	*Amazona finschi*		HP,CAM	[62]
Quailpox virus^1^	Scaled quail	*Callipepla squamata*	N	HP, CAM, EM	[117]
Sparrowpox virus^1^	Sparrows	*Passer domesticus*	N	HP, CAM, EM, PCR/Seq	[16,18]
Starlingpox virus^1^	Regal starling	*Cosmopsarus regius*	N	HP, CAM, EM	[118]
Turkeypox virus^1^	Turkey	*Meleagris gallopavo*	N	HP, CAM, EM, PCR	[119]
Crowpox virus^2^	Hawaiian crows	*Corvus hawaiiensis*	N	HP, CAM	[4]
Peacockpox virus^2^	Peacock	*Pavo cristatus*	N	HP, CAM	[120]
Penguinpox virus^2^	Penguin	*Spheniscus demersus*	N	CAM	[59]
Pigeonpox virus	Pigeon	*Palumbus palumbus*	N	HP, CAM, EM, PCR/Seq	[16,18]
Flamingopox virus	Flamingo	*Phoenicopterus roseus*	N	PCR/Seq	[121]
Partridgepox virus	Partridges	*Perdix perdix*	N	HP, EM, PCR/Seq	[122]
Sea Eaglepox virus	Sea eagle	*Haliaeetus albicilla*	N	HP, PCR/Seq	[123]
Great titpox virus	Great tit	*Parus major*	N	HP, EM, PCR/Seq	[18,124]
Curlewpox virus	Curlew	*Burhinus oedicnemus*	N	HP, EM, PCR/Seq	[125]
Common buzzardpox virus	Common buzzard	*Buteo buteo*	N	HP, CAM, EM, PCR/Seq	[126]
American crowpox virus	American crow	*Corvus brachyrhynchos*	N	HP	[127]
Ostrichpox virus	Ostrich	*Struthio camelus*	N	HP, CAM	[128,129]
Owlpox virus	Owl	*Strix varia*	N	HP, CAM	[130]
Goosepox virus	Canada Goose	*Branta canadensis*	N	HP, CAM	[15]
Magpiepox virus	Magpie	*Pica pica*	N	HP, CAM, EM	[16]
Mockingbirdpox virus	Mockingbird	*Mimus polyglottus*	N	HP, EM	[131]

Mode of infection: N, natural; E, experimental; HP, histopathological; CAM, chorioallantoic membrane; EM, electron microscopy; PCR, polymerase chain reaction; Seq, sequencing and GE, genetic and antigenic characterization.

^1^Currently classified by ICTV as members that belong to genus *Avipoxvirus*.

^2^Currently classified by ICTVas tentative members of genus *Avipoxvirus*.

^a^Complete genome sequences exists: Fowlpox virus FP9 (plaque-purified tissue culture-adapted attenuated European virus; accession number: AJ581527) and Fowlpox challenge virus (Animal Health Inspection Service Centre for Veterinary Biologicals, Ames Iowa; accession number: AF198100).

^b^Complete genome sequence exists: Canarypox virus strain Wheatley (American Type Culture Collection; ATCC VR-111).

## Definition

Avipoxviruses are large, oval-shaped enveloped viruses whose genome consists of double stranded DNA ranging in size from 260 to 365 kb [8]. Unlike most other DNA viruses, APVs replicate easily in the cytoplasm of infected avian cells which results in a characteristic cytopathic effect (CPE) 4 to 6 days post infection depending on the virus isolate [4]. APVs also multiply on the chorioallantoic membrane (CAM) of embryonated eggs, resulting in the formation of compact, proliferative pock lesions that are sometimes focal or diffuse [15]. However, some isolates, especially from the host species great tit (*Parus major*), have failed to multiply on CAM of chicken embryos [16]. APVs are the etiologic agent of disease characterized by skin lesions in both wild and domestic birds [4,5]. Histologically and ultrastructurally, APVs undergo morphologic stages that are similar to other chordopoxviruses, including the formation of intracytoplasmic inclusions bodies, a characteristic which has been observed in some epithelial and mononuclear cells of permissive hosts. APV particles can be detected and further characterized by use of transmission electron microscopy (TEM) [17,18].

## Classification

Great discoveries made in the mid-nineteenth century facilitated major advances in pox virology. Based on the report by Bollinger [5] on poxvirus infected cells in chickens, and subsequent work by Fenner and Burnet [19], APVs and other poxviruses were classified on the basis of original host, growth and morphological characteristics in the CAM of embryonated eggs or cell cultures and on clinical manifestations in different diseases of humans, birds and animals [20] rather than on genetic identity, which may provide both rapid and reliable virus identification [21-23]. These criteria have remained the basis for subsequent classification of APVs despite development of new molecular tools that have the capability of resolving the issue of species specificity of APV.

Members of the genus *Avipoxvirus *belong to the subfamily *Chordopoxvirinae *which shares several biological features with other poxviruses [7,8]. Currently, little is known of the number of species within the genus. While only ten strains have so far been identified and classified Worldwide as APV [1], avian poxvirus infections have been reported to affect a wide range of bird species [6]. These strains vary in virulence and host specificity, demonstrating an urgent need for further analyses and characterization of new isolates.

## Structure

**Figure 1:**
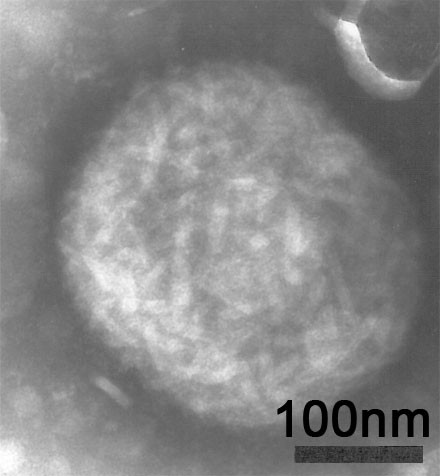
**Negative stain EM of a characteristic FWPV-particle propagated in baby hamster kidney (BHK) cells at 72 hour post infection**.

Avipoxviruses share several morphological, biochemical and physiochemical features with other poxviruses. Virus particles measure 270 × 350 nm and are composed of an electron dense, centrally located core and two lateral bodies that are visible in fixed and stained ultra-thin sections. In negative stained preparations, such as phosphotungstic acid (PTA) the membrane displays an outer-coat composed of a random arrangement of tubules [24]; Figure 1. APV particles have been shown to be resistant to ether, but sensitive to chloroform treatment [25], although resistance to both chloroform and ether have been reported for pigeonpox virus and two pigeonpox virus mutants [26].

## Genome

Avipoxviruses have low G+C content (30 to 40%) and consist of a single linear molecule of double-stranded DNA of between 260-365 kb. The central region of the genome is flanked by two identical inverted terminal repeats (ITRs) which are covalently linked by hairpin loops and contains several hundred closely spaced open reading frames [7]. The central region contains about 90-106 homologous genes that are involve in basic replication mechanisms, including viral transcription and RNA modification, viral DNA replication, and proteins involved in structure and assembly of intracellular mature virions and extracellular enveloped virions [8]. In general, genes located in this region have common molecular functions and are relatively conserved among poxviruses [8]. This is in contrast to the more variable, terminally located genes that have been shown to encode a diverse array of proteins involved in host range restriction [8].

Complete genomes of the two most studied APVs, FWPV US (FP-challenge virus; Animal Health Inspection Service Centre for Veterinary Biologicals, Ames Iowa, USA), FWPV-FP9 (a plaque-purified tissue culture-adapted attenuated European virus) and a CNPV virulent strain (Wheatley C93, American Type Culture Collection; ATCC VR-111) have been sequenced [7,8,27]. Although nucleotide and amino acids sequences for these two viruses are known, the functions of some putative genes and proteins remain to be fully assigned. Comparison of the FP9 strain with FWPV US revealed 118 differences; of which 71 genes were affected by deletion (26 of 1-9334 bp), insertion (15 of 1-108 bp), substitution, termination or frame-shift [27]. FP9 strain is a derivative of European FWPV HP1 which was obtained through over 400 passages in chicken embryo fibroblast (CEF). Analysis of FWPV HP1 sequences at the loci in which differences exist between FP9 and FWPV US show that 68 of 118 loci differ from the FWPV US, but were identical to FP9. This thus indicate that more than half of the differences between the two geographic FWPV lineages represented differences between the parent virulent viruses FWPV HP1 and FWPV US [27]. Further comparison of molecular data; show that FWPV and CNPV share high amino-acid identity, significant gene-sequence rearrangements, deletions and insertions [8]. The CNPV genome is about 80-100 kbp larger than the FWPV genomes. Both FWPV and CNPV express cellular gene homologues with immunomodulatory functions, which might be responsible for their different virulence and host-range [8], but CNPV shows a broader tissue tropism in permissive avian hosts [17] than FWPV. CNPV has additional sequence of over 75 kbp, 39 genes lacking in FWPV homologues and approximately 47% amino-acid divergence [8]. These divergences are primarily found in the terminal non-conserved regions [7,8]. Genes located in the non conserved regions are more prone to mutation and recombination and are implicated in host range, immunomodulation and pathogenesis [28], and may be responsible in some aspects of cell and/or tissue tropism or perform other cellular functions [8].

Virulence genes are generally of non-conserved nature and influence the pathological profile of viruses in an infected host. These genes are important in viral evolution and have been used in studies to provide insight into how some poxviruses evolve strategies to ensure their replication [29]. Many of these strategies can be traced back to discoveries made with knockout (KO) viruses, in which a targeted disruption of a specific viral gene produced phenotypic changes reflective of the normal biological function of its protein product. Deletions of some non-conserved genes have also resulted in conditional replication defects in specific cell types [30], such as the demonstration of spontaneous deletion of host range genes of vaccinia virus resulting in the compromised growth in the mammalian cell [31]. The K1L and C7L genes of vaccinia virus have been shown to be essential for completion of the replication cycle of vaccinia virus in human cells [32,33]. In a knockout experiment, vaccina virus was unable to complete its replication cycle in Chinese hamster ovary (CHO) cells, since replication was aborted shortly after virus binding and entry, at the stage of intermediate gene expression [34]. But the insertion of another host range gene, CHOhr from cowpox virus into vaccinia virus allowed vaccinia virus to grow in CHO cells in which they are normally restricted [35-37]. Through use of these techniques, we now have a better understanding of the biology of vaccinia and other poxviruses, including their host range restriction. Although, some major advances have been made in genome sequencing and *in vitro *characterization of APVs [7,8,38,39], studies on APV host range genes are scarce. A wide array of gene homologues with likely host range functions such as NK-cell receptors, chemokines, serine protease inhibitors and homologues of genes involved in apoptosis, cell growth, tissue tropism and avian host range, have been identified in APVs, which suggests significant viral adaptation in the avian host [7]. Molecular knockout studies that target identification and further characterization of viral genes involved in regulation of cell proliferation, chromatin remodelling, virulence and apoptosis, in different APV-infected mammalian and avian cells are needed to better understand the tissue tropism and host range characteristics of APVs, including the abortive infection in mammalian cells.

## Host-virus interaction

Compared to other poxviruses, such as vaccinia virus, mechanisms that account for APV pathogenesis is poorly understood. APVs have evolved a variety of elegant mechanisms to deliver their genes and accessory proteins into host cells. Like many other DNA viruses, APV probably devotes much of its genes to allow it to evade host immune responses. Such viral genes commonly encode proteins that are critical for the virus to undergo molecular transformation that leads to successful membrane fusion, penetration and intracellular transport. These includes genes that encodes proteins which act on early innate pathways such as pathways involving interferon [40], pattern recognition receptors as Toll-like receptor (TLR) [41], chemokines [42] and cytokines [43], as well as pathways that act on subsequent adaptive responses [44,45]. The infection of a cell by a virus is a complex process, during which the virus must overcome several host factors restriction points and the host immune response. Host protein interaction networks and biochemical pathways are in most cases altered by the viral proteins that free the virus from normal cellular controls and allow nucleotide metabolism in cells that have shut down DNA synthesis [46]. Hence, understanding viral protein functions and their interactions with host proteins is a prerequisite, not only to understand the infection biology of the virus-host system in question, but also for the rational development of target vaccines, based on specific antigens and possibly immunomodulatory factors, as well as antiviral compounds.

### Replication

Since the first isolation of APVs in cell culture, these viruses have been recognized as highly host specific. They are believed to replicate only in avian cells, notably chicken embryo fibroblasts (CEF; American Type Culture Collection; ATCC, Rockville, Maryland, USA; CRL-1590) [47]. CEF cells have a good split ratio compared to other cell lines, and are thus useful for large-scale propagation of virus, such as antigen production for vaccines or as a diagnostic tool. APVs have also been shown to replicate in chicken embryo kidney, chicken embryo dermis [48,49] and quail cell lines, such as QT-35, although the presence of viable endogenous herpesvirus and Marek's disease virus (MDV) in QT-35 cells, limits their use for preparation of vaccines [4,50]. APV have been isolated once from a mammal. In 1969, viable FWPV was isolated from a terminally ill rhinoceros [51]. The isolate was identified as atypical FWPV, based on pathological, virological and serological characteristics [51]. Nelson (1941) [52] reported mild pathology in mice following intranasal inoculation with FWPV, with no virus replication. Recent studies have also shown replication of APV in mammalian cell cultures, such as embryonic bovine tracheal cells [53] and baby hamster kidney cells [54] that are defined by the presence of infectious viral particles and CPE. These studies raise questions about the species specificity and mechanisms that restrict these viruses to certain hosts, and challenge the hypothesis that APV cannot undergo a full replication cycle in mammalian cells.

### Morphogenesis

The cellular entry and exit of APV is complicated by the existence of at least two distinct forms of virus that can productively infect cells, namely the intracellular mature virus (IMV) and the extracellular enveloped virus (EEV). These two forms are surrounded by different lipid membranes and surface proteins that are yet to be fully characterized.

**Figure 2:**
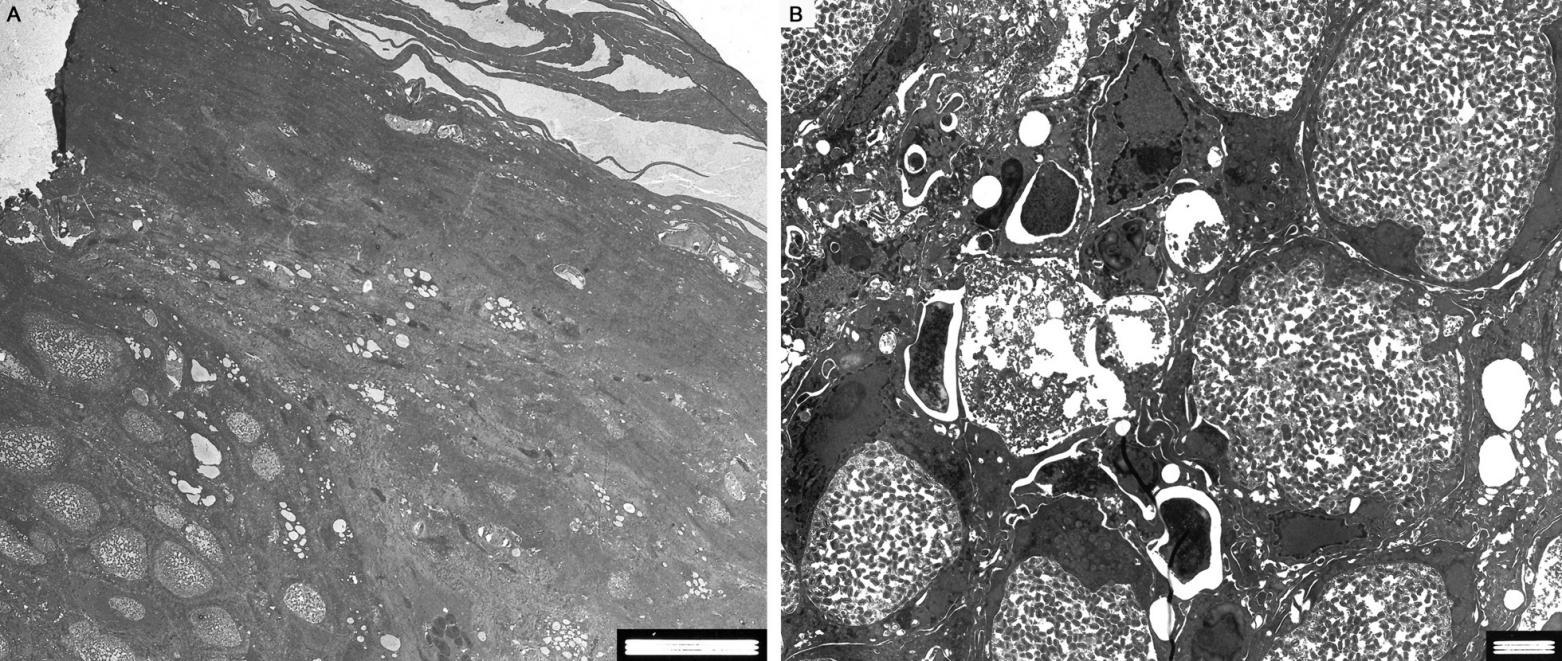
**A and B Transmission electron microscopy (TEM) of FWPV-infected (A) bird skin tissue showing outer layers of epidermis, with typical inclusion bodies (Bollinger bodies) in the dermis (bar = 20 μm) and (B) cells with characteristic intracytoplasmic inclusion bodies containing viral particles (bar = 2 μm)**.

After virus binding to cellular membranes, a fusion step, which is generally poorly understood, results in the release of the virion core into the cytoplasm of the cell [39]. The released core, which contains the endogenous RNA polymerase and transcription factors, initiates the first wave of early viral gene transcription by synthesizing viral mRNA under the control of early viral promoters. This is followed by the uncoating stage, the release of viral DNA into the cytoplasm where it serves as a precursor for viral DNA replication as well as the source of intermediate and late viral gene transcription. As a late viral gene product accumulates, the virus undergoes assembly and morphogenesis of infectious virus particles. During morphogenesis, APVs induce the formation of inclusion bodies in the cytoplasm of infected cells (Figure 2A and 2B). The inclusions, which may also be termed viral factories, viroplasms, or viral replication complexes, are generally believed to be the sites of active viral replication and particle assembly within infected cells [17]. One model for the function of viral inclusion bodies is that they act to concentrate and sequester proteins, nucleic acids, and other small molecules essential for viral processes.

**Figure 3:**
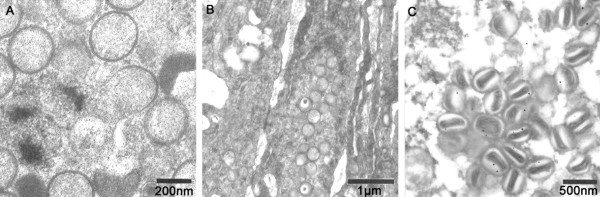
**A, B and C Virion morphology of avipoxviruses in chicken embryo fibroblast cells (CEF)**. (A) Electron microscopy showing crescent-shaped structures consisting of a membrane with spicules on the convex surface, (B) spherical non-infectious immature viruses (IV) which give rise to formation of (C) intracellular mature virions (IMV) by a series of maturation steps.

In permissive cells, the first viral structures detectable by electron microscopy are the crescent-shaped forms (Figure 3A), consisting of a membrane with spicules on the convex surface [39]. These structures develop into non-infectious spherical immature viruses (IV) (Figure 3B) from which the intracellular mature virus (IMV) is formed by a series of maturation steps (Figure 3C). The IMV represents the majority of infectious progeny from each infected cell [17,39,55]. There are three possible mechanisms by which poxviruses are released from host cells depending on the strain of virus, cell type and the post-infection time [54-56]. They can be released by cytolysis, in which case IMV are released when the cell undergoes lysis as a result of CPE at the advanced stage of infection. It can also be released via virus-induced exocytosis. The third way of release is by budding, in which case IMV migrates out of the virus factory through the plasma membrane. Budding is shown to be the main exit route for APV [39] in contrast to the orthopoxviruses which exit by exocytosis of intracellular enveloped virus (IEV) [57]. These exit processes all result in the acquisition of an additional double membrane [39,55]. In non-permissive African green monkey cells (CV-1, Vero) and in a human cell line (MRC-5), there is a blockade of the APV morphogenesis cycle. This occurs in steps following the formation of immature virus and is shown to be devoid of an alteration in early gene expression [47,58,59], indicating that this blockade may not be associated with cell receptors.

Poxvirus tropism may not be dependent upon specific cell surface receptors, but rather upon the ability of a given cell to provide intracellular complementing factors needed for productive virus replication, and on the ability of the specific virus to successfully manipulate intracellular signaling networks that regulate cellular antiviral processes following virus entry [28]. APVs have large genomes that would enable them to express unique collections of viral proteins that function as host range factors, which specifically target and manipulate host signaling pathways to establish optimal cellular conditions for viral replication. However, in some cells, especially mammalian cells, APV replication is blocked. This may be due to the ability of APV to specifically activate signalling pathways or mediators, for example interferon pathways, in those cells. The role of mediators and immunopathology of APV is complex and not well understood. However, considering the numerous steps involved in APV morphogenesis, it is relevant to note that these viruses induce several mediators that allow them to survive and interact with the host cells. Some potential mediators have been identified [7] and are awaiting proper functional characterization. These molecules alone may not fully explain the events that have been documented in mammalian cells that have supported the replication of APV [54]. Hence, identification of new mediators that are up or down-regulated in response to APV infected mammalian and avian cells could help advance our knowledge of immune responses against APV and the related immune-mediated pathology and cell tropism. It would be of vital importance to investigate these characteristics further, especially for the cell types that were recently shown to support APV replication [53,54].

## Pathogenicity

APV infections are associated with significant levels of morbidity and mortality in domestic and wild bird populations [6,60]. Most of the investigations and reported cases are based on single APV isolates, which makes it difficult to address the pathogenicity of different APVs in different bird species. Chickens are commonly used to determine the pathogenicity of new isolates, but chickens may not be the ideal host, since APVs from wild birds may not multiply in chickens. In an attempt to identify and characterize the pathogenicity of APVs, Tripathy and others [4] found that wild isolates of Hawaiian crowpox virus had a generally mild pathogenicity in domestic chickens, characterized by relatively minor lesions of short duration at the sites of inoculation, which were in contrast to the general ability of FWPV strains to produce extensive proliferative lesions [4]. In another experimental study, two APV isolates obtained from endangered Hawaiian wild birds, the Hawaiian Goose (*Branta sandvicensis*) and the Palila (*Loxioides bailleui*), were compared with FWPV in specific-pathogen-free chickens. Immune responses were measured by ELISA before and after immunization with Hawaiian APVs and after challenge with FWPV. Both isolates from Hawaiian birds developed only a localized lesion of short duration at the site of inoculation in chickens and did not provide protection against subsequent challenge with virulent FWPV, in which severe lesions were observed. In contrast to high antibody response in chickens immunized with FWPV, birds immunized with either of the two Hawaiian isolates developed low to moderate antibody responses against viral antigens [61]. Pathogenicity studies of APVs in parrots [62], turkeys, pigeons and canaries have also been reported. Canaries were highly susceptible to CNPV, but showed resistance to turkeypox virus, FWPV and pigeonpox virus [4,63,64]. A poxvirus from a Canada goose (*Branta canadensis*) was transmissible to domestic goose, but not to chickens or domestic ducks [15]. Pigeonpox virus produced mild infection in chickens and turkeys, but was more pathogenic for pigeons [62]. Poxvirus isolates from magpies (*Pica pica*) and great tits (*Parus major*) did not infect young chickens [16], however, poxvirus isolated from black-backed magpie *(Gymnorhina tibicen) *produced lesions in chickens. These studies were based on clinical manifestations in the chickens and suggest host specificity and pathogenicity.

Despite the worldwide prevalence of APV infections, experimental infection studies in birds using APVs have centred on relatively few viral isolates. Analyses of variation have essentially focused on a FWPV strain termed the prototype, while a minority of experimental studies have been reported on CNPV, quailpox, juncopox, and pigeonpox virus isolates [4,48,49]. In fact, in the last twenty years, approximately 50% of published studies on APVs have been on the FWPV isolate directly (based on a PubMed search on APVs). The important nature of APVs which has been used successfully for vaccine development mandates that a larger pool of viral strains should be analyzed both for consideration of pathogenesis and determination of immune correlates of protection.

### Antigenic and genetic variability among APVs

Our present understanding of the antigenic variation of APVs has been based on a limited number of virus isolates in assays that includes complement-fixation, passive hemagglutination, agar-gel precipitation, immunoperoxidase, virus neutralisation and immunofluorescence [48,49,65]. In addition to the immunological assays, variation of APVs has also been addressed through genetic assays, such as restriction enzyme analysis. Genomes of FWPV and quailpox virus isolates were compared by using *Bam*HI, *Eco*RI, and *Hind*III endonucleases and distinct fragment patterns were observed between the isolates. The patterns of three quailpox virus isolates were similar to each other with a high proportion of co-migrating fragments. However, when immunogenic proteins of three FWPVs, two quailpox viruses, a juncopox virus, and a pigeonpox virus isolates were examined by immunoblotting, shared as well as unique antigens were detected. The greatest disparity was observed between quailpox virus and FWPV [48,49], indicating extensive variation between the quailpox virus and FWPV, which would predict differences in immunogenicity and antigenicity, including neutralization sensitivity. Nucleotide sequence based studies for rapid identification of poxvirus species by PCR with specific primers and hybridisation are well established [21]. These approaches have concentrated on single genes or portions of genes that exhibits variations in their sequence and are important for quick analysis of genetic variability [22].

### Phylogeny

Understanding the phylogenetics of APVs is essential to the understanding of host specificity and virulence, but also to provide insights into the variation of different viruses. Although the complete genome sequences of FWPV and CNPV are available [7,8], little is known about APV phylogeny. This is probably because of the difficulty in identifying pan-genus or species-specific PCR primers that can be used to amplify different genes. The most common PCR locus used until now has been the P4b locus [21]. Recent phylogenetic studies of APV isolates based on this locus [22,23] indicated that most isolates clustered around either CNPV or FWPV, while another study based on the same locus demonstrated a third cluster, from psittacine birds [66]. Amano and coworkers [38] showed that the CNPV thymidine kinase locus was highly diverged from that of FWPV. The extent of this divergence was further illustrated by the fact that the amino acid similarity between CNPV and FWPV orthologue P4b was only 64.2% [23,38]. A recent study, based on three different genes including the P4b, revealed that penguinpox virus, isolated from lesions around the eyes of African penguins (*Spheniscus demersus*), was most closely related to turkeypox virus, ostrichpox virus and pigeonpox virus [67].

## Diseases

**Figure 4:**
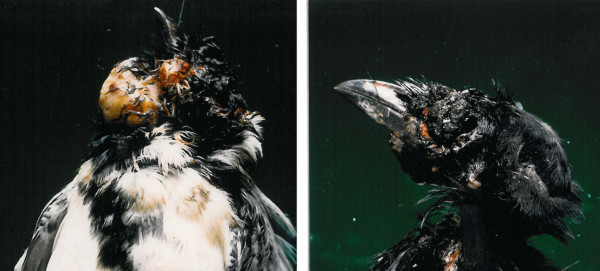
**Avipoxvirus infection of a great tit (*Parus major*) (left) and a common magpie (*Pica pica*) (right)**. The protecting feather coat is destroyed. Proliferative lesions and crust are seen, with secondary bacterial infection (Photo: Gunnar Holt, National Veterinary Institute, Oslo, Norway).

During avipox outbreaks, mortality can reach 80 to 100% in canaries and other finches. This is in contrast to a generally lower mortality seen in chicken and turkey [60]. Transmission of virus can occur through a break in the skin or, more commonly, when vectored by biting insect such as mosquitoes and mites [68]. Aerosols generated from infected birds, or the ingestion of contaminated food or water have also been implicated as a source of transmission [69]. The disease is most commonly characterized by cutaneous proliferative lesions consisting of epithelial hyperplasia of the epidermis that resulting in proliferative, wart-like projections. They are primarily confined to unfeathered parts of the body, such as legs, feet, eyelids and the base of the beak (Figure 4). Scars are usually visible after recovery and healing of skin lesions. The mortality in wild birds is usually low, depending on the number and size of the proliferative lesions. However, if infection occurs in feather-free areas of the skin, with secondary bacterial infection, mortality may be high. The other and less common form of APV infections is the diphtheritic or wet form [70] which occurs as fibrino-necrotic and proliferative lesions in the mucosa of the digestive and upper-respiratory tracts, and generally has a higher mortality than the cutaneous form [60]. In some instances, birds display both cutaneous and diphtheritic forms and in those cases, mortality rates are often higher compared to the cutaneous form alone. Despite the variety of hosts and virus strains, associated pathology remains the same in infected domestic birds, although clinical signs vary depending on the virulence of the virus, susceptibility of the host, distribution and type of lesions [60]. There exist a relationship between FWPV and the avian retrovirus, reticuloendotheliosis virus (REV) (see section on APVs and REV). However, the possible roles that simultaneous REV infection arising from the provirus integration into the FWPV genome might play in the expression FWPV during disease outbreak remain unresolved. It is well known that REV infection leads to immunosuppresion [71] in affected birds. Thus, it is plausible to suggest that the presence of REV in FWPV infection may exacerbate disease progression. In spite of the fact that some mammalian cell lines seems to be able to support the replication of APVs, there is no evidence that APVs have caused clinical disease in humans, in contrast to what is known for other poxviruses, such as several parapox and orthopoxviruses.

## Diagnosis of APV infections

### Clinical diagnosis

Clinical features of infected birds show multiple skin lesions varying from papules to nodules. Gross lesions in both the cutaneous and the diphtheritic forms, seen on birds and during necropsy, are usually sufficient to suspect APV infection [60]. However, these signs are sometimes not sufficient for definitive diagnoses of APV infection as other agents, such as papilloma virus, scaly leg mites [72] and mycotoxins may produce similar lesions in the skin [60], and conditions like candidiasis, capillariasis and trichomoniasis may give lesions in the oral cavity similar to the diphtheritic form of APV infection [73]. It is therefore crucial to secure samples and confirm the viral etiology of the condition.

### Laboratory diagnosis

#### Histopathology and electron microscopy

Suspicion of clinical signs of APV infection can if possible be supported by necropsy, especially if the oral cavities to reveal the diphtheritic form. Further, histopathology on tissue sections using the classic Wright's Giemsa stain may reveal typical large, solid or ring-like, eosinophilic intracytoplasmic inclusions known as Bollinger bodies [5]; Figure 2A and 2B. Transmission electron microscopy (TEM) may also reveal definite proof of APV infection, demonstrating the typical APV particles within inclusion bodies. APV identification may also be carried out by negative staining electron microscopy with 2% phosphotungstic acid (PTA) on infected cells (Figure 1). This method has typically been used by national reference or research laboratories to identify APV [18].

#### Virus isolation

Demonstration of infectious virus by inoculation of homogenates of clinical samples of typical APV skin lesions onto the CAM of embryonated hen's eggs is the gold standard method for diagnosis of APV, although some strains of APV do not grow readily on chicken embryos [16]. Eggs are first swabbed with 70% alcohol and a pore is made in an area over the air-cell and another one on the other side of the egg to make a false air sac and lower the CAM by negative pressure using a rubber bulb. Inoculation of infectious samples by the CAM route is performed with sterile disposable 1 mL syringe with approximately 0.1-0.2 mL of inoculum. Eggs are incubated at 37°C for 5 days with daily candling to check for embryo death. Pock lesions measuring in size 0.5-1.5 mm are observed on the membrane 3-5 days after inoculation, depending on the virulence of the virus [15,16]. Another method of isolation of APV requires the excision and homogenization of clinical skin lesions and inoculation of a homogenate supernatant onto a permissive cell culture, such as CEF cells. This results in the formation of CPE within 4-6 days post inoculation, depending on the virus isolate and on the multiplicity of infection (MOI) [4].

#### Molecular techniques for detection and characterization

APV are increasingly being detected and characterized by PCR, Restriction fragment length polymorphism (RFLP), Southern blot hybridization, and cycle sequencing, directed at specific genes such as the 4b core protein gene [22,23]. PCR allows for sensitive and specific detection of viral nucleic acids and has been shown to increase the diagnostic sensitivity for many viral pathogens when compared to culture. A PCR amplicon sequence allows a rapid search for homologous sequences in gene databases, to verify and identify the virus in question and to address phylogenetic relationships. Detection by real-time PCR has been used to identify recombinant APV from individual plaques [74]. This method eliminates the need for amplification and hybridization from the transient dominant protocol and results in significant savings of time at each round of plaque purification [74].

#### Serological assays

The conventional serological techniques of passive neutralization and agar-gel immunodiffusion are in continued global use for surveillance and disease control efforts in domestic poultry species [75,76], despite the availability of modern molecular and immunoassay techniques. The tests are time consuming, especially when carried out with large numbers of sera, and sensitivity appears to be low when compared with other detection method, such as enzyme linked immunosorbent assay (ELISA) [77]. ELISA has been described as a non-species specific test approach for birds [78]. It is a faster and easier method to detect antibodies against APV, particularly when large numbers of sera are to be tested. The technique is also more sensitive than the neutralization test [18,78]. ELISA protocols have also been developed and used to test the efficacy of FWPV vaccines in commercial and wild bird species where agar-gel immunodiffusion is ineffective due to lack of precipitating antibodies [61,79].

## Prevention and treatment

The challenges of controlling APV disease in poultry are driven by economics, and require strategies that keep cost low while maintaining treatment efficacy. Prophylaxis can be achieved by vaccination [39]. Doyle [80] reported the use of live FWPV or Pigeonpox virus for vaccination against APV infection. Since then, recombinant and live modified vaccines have been developed and used to prevent APV infections in chickens, pigeons, turkeys and quails [79,81,82]; Table 2. These vaccines are very effective and have undoubtedly contributed immensely to the prevention of the disease in commercial poultry farming [47,81]. Since different APVs are isolated from a wide range of bird species and since only a few isolates have been characterized, development of a taxon-specific vaccine, directed to all species, has been difficult. Thus, available vaccines are often applied on the basis of experimentation, and more knowledge of molecular biology, pathology and epidemiology of these viruses is necessary to develop vaccines that effectively can protect a range of bird species. As in most viral infections, there is no specific treatment for avian poxvirus infections in birds [39,83]. Available treatments include the use of iodine-glycerin application on proliferating skin lesions to aid healing [84], antibiotics to control secondary bacterial infections and vitamin A to aid healing [85].

**Table 2:** Avipoxvirus vector-based vaccines licensed for commercial veterinary use

Recombinant viral vector	Inserts	Targeted Pathogen	Species	Distributor	Country	References
ALVAC	G	Rabies virus	Cats	Merial, Inc.	USA, Canada	[109]
ALVAC	HA and F	Canine distemper virus	Dogs, ferrets	Merial, Inc.	USA, Argentina, Brazil, Colombia, Canada, Uruguay	[132]
ALVAC	PrM-E	West-Nile virus	Horses	Merial, Inc.	USA, Canada	[133,134]
ALVAC	Env, Gag/pol	Feline leukaemia virus	Cats	Merial, Inc.	Europe, USA, Canada	[135-137]
FWPV	H5 HA	Avian influenza virus	Chickens	Merial, Inc.	USA, Canada	[110]
ALVAC (plus tetanus toxoid and Carbopol adjuvant)	HA	Equine influenza virus	Horses	Merial, Inc.	Europe, USA	[111]
FWPV	HN and F	Newcastle disease virus	Chickens	Biomune	USA	[13,138]
FWPV- Laryngotracheitis Vaccine	LT+AE	Infectious Laryngotracheitis virus	Chickens	Biomune Co. (Lenexa, KS, USA)	USA	[139]
FWPV- Mycoplasma gallisepticum Vaccine	MG+AE	Avian Encephalomyelitis and *Mycoplasma gallisepticum*	Chickens	Biomune Co.	USA	[140]

**Note**: HA from influenza virus H5; HA, hemagglutinin; HN, haemagglutinin-neuraminidase protein; ALVAC, attenuated canarypox virus; FWPV, fowlpox virus; F, fusion protein; G, glycoprotein G; Env, envelope glycoprotein; Gag, group specific antigen; Pro, protease; prM-E, pre-membrane and envelope proteins; MG+AE, *Mycoplasma gallisepticum *+ Avian Encephalomyelitis; LT+AE, Laryngotracheitis virus+Avian Encephalomyelitis.

## Avipoxviruses and reticuloendotheliosis virus (REV)

In the poultry industry, prophylactic measures against FWPV are achieved primarily by vaccination with live FWPV or antigenically similar pigeonpox virus strains produced in CEF cells [60]. In the past two decades, numerous outbreaks have been reported in vaccinated flocks, suggesting that vaccines used against the disease were not effective. In the United States a commercial FWPV vaccine was shown to be contaminated with REV and caused lymphoma among broiler chickens [86]. It has been shown that sequences of REV have been integrated into the DNA of FWPV vaccines as well as in field FWPV isolates [81,87-90]. The integration site is constant, while the size of the integrated fragments differs between various isolates and strains. Two different types of integrated sequences are reported; long terminal repeats (LTRs) with size of approximately 200 to 600 bp and the near-full-length REV provirus of about 800 bp [87,90,91]. Most vaccine strains carry only an LTR remnant while most FWPV field isolates carry the near-full-length provirus. Singh and others [81], however, detected REV LTRs of various lengths in the genome of two commercial FWPV vaccine strains and four field isolates, while several studies have shown that the source of REV infection was REV-contaminated FWPV [86,92-94] and herpesvirus of turkeys vaccines [92,94-97]. Reticuloendotheliosis is a tumorigenic and immunosupressive disease. REV strains have been reported to cause diseases characterized by chronic lymphoma, non-neoplastic lesions and a runting-stunting syndrome in chickens, turkeys, and quails [98,99]. REV are group of avian retroviruses and representatives include the defective REV-T and the non-defective REV-A, spleen necrosis virus (SNV), duck infectious anemia virus, and chick syncytial virus (CSV) [98]. The presence of REV in FWPV vaccines and the failure of currently used FWPV vaccines to evoke high level immunological protection against field challenge of FWPVs are of major concern to the poultry industry [100], which emphasizes the need for research into alternative vaccines.

## Avipoxviruses as vaccine vectors

In 1796 Edward Jenner [101] published his landmark findings that vaccination of humans with cowpox virus could prevent infection with variola virus, the causative agent of smallpox [101,102]. This traditional vaccine technology, based on live viruses and immunological cross protection, has given rise to a wide range of effective vaccines against a wide variety of infectious agents, both in veterinary and human medicine. However, the emergence of new deadly human pathogens and cancers, have proven less amenable to the application of traditional vaccine platforms, indicating the need for new approaches. The use of a live virus vector represent an attractive way to deliver and present vaccine antigens that may offer advantages over traditional platforms, by improving the quality and strength of the immune response, such as in the case of HIV-1 where two different strains of vaccinia virus have been used as vectors. The NYVAC vector has been shown to induce the CD4^+ ^T cell-dominant response, whereas modified vaccinia virus Ankara (MVA) induces a stronger CD8^+ ^T cell response with accompanying CD4^+ ^T cell responses that are required for protection [103]. Although this assertion remains unproven (there are to date no virally vectored vaccines licensed for human use), virally vectored vaccines offer an avenue of possibilities, either as homologous regimens, or as heterologous (prime-boost) regimens in which different serotypes of a given vector, different vectors or vectors and traditional technologies such as recombinant protein in adjuvant are administered sequentially.

Currently, representatives of a wide range of virus families are under intensive development as vaccine vectors for human or veterinary use. Of these, FWPV and CNPV appear to be of great interest as vectors, and some veterinary APV-vectored vaccines are already licensed and in commercial use in North America, South America and Europe (Table 2). The most important characteristics of APVs as vaccine vectors are that unlike most other DNA viruses, APV replicate in the cytoplasm of the infected cell and enzymatic functions used for transcription and replication are provided by the virus itself. This has several consequences regarding the use of these viruses as vaccine vectors. For example, APV promoters must be used for efficient transcription of recombinant genes and as APV transcripts are not spliced, genes cloned into APV vectors cannot contain introns [70,104]. Other reasons include (1) their ability to accommodate and effectively express large amounts of foreign DNA or multiple genes that encode antigens [47], (2) their inability to conduct a full replication cycle in non-avian species [105-107], (3) antisera against orthopoxviruses do not neutralize APV and thus, prior exposure to vaccinia virus (i.e., vaccination against smallpox) and exposure to other orthopoxviruses such as cowpox virus does not impact the immunogenicity of FWPV and CNPV vectors, and (4) the fact that FWPV and CNPV do not elicit high levels of neutralizing antibodies, which means that their vectors can be used multiple times without the diminished potency usually seen with repeated use of vaccinia virus vectors. This attribute is of crucial importance for a therapeutic vaccine, requiring repeated booster shots [108].

APV-vectored vaccines have been used as vaccines against several animal infections including West Nile virus (WNV), canine distemper virus, feline leukemia virus, rabies virus, and equine influenza virus [109]; Table 2. Notably among them is the Trovac AI H5, a recombinant FWPV that express the H5 antigen of avian influenza virus. This product has had a conditional license for emergency use for chickens in the United States since 1998 and has been widely used in Central America, with over 2 billion doses administered [110]. ALVAC vectored vaccines have recently been registered for veterinary use in the European Union (Proteq-Flu) [111] and the United States (Recombitek). The equine influenza virus vaccine with CNPV vector expresses the hemagglutinin genes of the H3N8 Newmarket and Kentucky strains and contains a polymer adjuvant (Carbopol; Merial Ltd.). With the induction of both cell-mediated and humoral immunity, it is claimed that the vaccine produced sterile immunity 2 weeks after the second of two doses. The new vaccine is also designed to protect horses against the highly virulent N/5/03 American strain of equine influenza virus and to prevent the virus from spreading through the elimination of viral shedding.

Despite these notable advances in APV-vectored vaccine development, the list of licensed viral vectored vaccines for human medicine is short, with only a few vaccines that have entered clinical trials [112]; Table 3. This may be in part owing to stringent safety requirements that must be met for viruses, that in their natural state have the potential to be human pathogens, to be used as viral vaccine vectors that may replicate *in vivo *in a manner similar to their wild-type parental viruses. Another reason may be fear of risk of spontaneous recombination between virus vectors and naturally occurring viral relatives in the ecosystems in which the vaccine is used. Even if APVs are not generally expected to replicate in mammals, the vaccine vectors may reach bird populations via animal populations. It is also possible that the vector, through spontaneous recombination and mutation events, may restore its replication competence. To cater for this, the aim during design and development of a virus vector is always to introduce at least two gene deletions crucial for viral to undergo a full replication cycle to assure a very low probability that replication competence could be restored. To our knowledge, such reversions have not been identified in clinical trials of APV-vectored vaccines. In addition to concerns regarding reversion or recombination, another safety signal was recently identified in an *in vitro *experiment that showed APV replication in cell clones derived from embryonic bovine trachea [53] and Syrian baby hamster kidney (BHK) cells. In this experiment, infectious IMV was observed; indicating complete virus replication had taken place [54]. These findings are in contrast to the general dogma that APVs are restricted to infection of cells of avian origin, and are an indication that there is still more to learn about the replication mechanisms and virus-host interactions of these viruses, including evasion of immune responses, cell tropism and host range mechanisms.

**Table 3:** Avipoxvirus vector based vaccines and prime-boost immunization regimes in clinical development for human use

Recombinant viral vector	Targeted pathogen/disease	Inserts	Vaccine details	Developer/Sponsors	Clinical phase	References
ALVAC containing the gene encoding HIV-1 gp160, and protein gp120	HIV-1	gp160 and gp120	ALVAC-HIV vCP1521 prime and AIDSVAX-gp120 subtype B/E boost	U.S. Army Medical Research and Materiel Command	III	[141]
ALVAC-HIV (vCP1452) and LIPO-5+vCP1452	HIV-1	LIPO-5	ALVAC-HIV (vCP1452) LIPO-5	NIH	I/II	[142]
VACV and ALVAC or FWPV with or without combination therapy	Cancer	CEA (pancarcinoma)	TRICOM vectors co-express B7.1, ICAM1 and LFA3	NIH	I/II	[143]
FWPV (FP9)	Malaria	Circumsporozoite (CSP) protein	Attenuated FP9 and Circumsporozoite (CSP) protein	Gates Malaria Partnership	I	[144]

HIV-1, human immunodeficiency virus 1; ALVAC, attenuated canarypox virus; gp160, glycoprotein 160; gp120, glycoprotein 120; VACV, vaccinia virus; FWPV, fowlpox virus; CEA, carcinoembryonic antigen; TRICOM, triad of costimulatory molecules; ICAM1, intercellular adhesion molecule 1; LFA3, lymphocyte function-associated antigen 3; LIPO-5, Lipopeptide 5; CSP, circumsporozoite protein; FP9, attenuated fowlpox virus 9; NIH, US National Institutes of Health.

## Conclusions

APVs cause disease of economical importance for the poultry industry, and also in pet and wild birds. Thus, prophylactic measures, such as vaccination, will always be required, and there is a need for more efficient and safe vaccines. One promising approach is the use of APVs as vectors for recombinant vaccines, increasing the efficacy and avoiding the potential contamination with REV and other agents. Many recombinant APV constructs are already licensed for use in veterinary medicine, and a range of vaccine candidates are currently being tested for use in vaccines against numerous infectious diseases in animals and man. Thus, it is likely that recombinant APV-vectored vaccines in the near future will also be used against human diseases. APVs have many advantages as vaccine vectors, including a large genome which allows for the inclusion of many heterologous genes, such as genes coding for antigens, cytokines and other immuno-modulating factors. The major safety argument for using APVs rather than vaccinia virus or other mammalian viruses as vectors, is that APVs are not zoonotic and are not able to conduct a full replication cycle in mammals. However, it was recently shown that FWPV was able to replicate and produce progeny virions in some established mammalian cell lines. This illustrates the fact that general knowledge of APVs is scarce. Indeed, only a few isolates have been characterized and classified. New molecular tools have led to a greater resolution of factors and mechanisms that restrict viruses to certain hosts, for example HIV and SARS. Mechanisms of host restriction, pathogenicity, host immunity and viral immune evasion strategies are of crucial importance regarding use of APVs as vectors in multispecies-targeted vaccines. A good understanding of the molecular properties of APVs underpins the development of safe APV-vectored vaccines.

## Abbreviations

ALVAC: attenuated canarypox virus CNPV; APVs: Avipoxviruses; ATCC: American Type Culture Collection; BHK: Baby hamster kidney; CAM: chorioallantoic membrane; CEA: carcinoembryonic antigen; CEF: chicken embryo fibroblast; CHO: Chinese hamster ovary; CPE: cytopathic effect; CSP: circumsporozoite protein; EEV: extracellular enveloped virus; ELISA: enzyme linked immunosorbent assay; Env: envelope glycoprotein; F: fusion protein; FP9: attenuated fowlpox virus 9; FWPV: fowlpox virus; G: glycoprotein G; Gag: group specific antigen; gp120: glycoprotein 120; gp160: glycoprotein 160; HA: hemagglutinin; HA5: Hemagglutinin from influenza virus H5; HIV-1: human immunodeficiency virus 1; HN: haemagglutinin-neuraminidase protein; ICAM1: intercellular adhesion molecule 1; IEV: intracellular enveloped virus; IMV: intracellular mature virus; ITRs: inverted terminal repeats; IV: immature virus; LFA3: lymphocyte function-associated antigen 3; LIPO-5: Lipopeptide 5; MDV: Marek's disease virus; MOI: multiplication of infection; NIH: US National Institutes of Health; PCR: polymerase chain reaction; prM-E: pre-membrane and envelope proteins; Pro: protease; PTA: phosphotungstic acid; REV: Reticuloendotheliosis virus; RFLP: restriction fragment length polymorphism; TEM: transmission electron microscopy; TLR: Toll-like receptor; TRICOM: triad of costimulatory molecules; VACV: vaccinia virus; WNV: West Nile virus;

## Competing interests

The authors declare that they have no competing interests.

## Authors' contributions

SCW and MT contributed equally in drafting and reviewing the manuscript. All authors have read and approved the final manuscript.
